# A single-set functional training program increases muscle power, improves functional fitness, and reduces pro-inflammatory cytokines in postmenopausal women: A randomized clinical trial

**DOI:** 10.3389/fphys.2023.1054424

**Published:** 2023-03-22

**Authors:** Jackson Neris de Souza Rocha, Alan Bruno Silva Vasconcelos, José Carlos Aragão-Santos, Antônio Gomes de Resende–Neto, Marcos Raphael Pereira Monteiro, Albernon Costa Nogueira, Alan Pantoja Cardoso, Cristiane Bani Corrêa, Tatiana Rodrigues de Moura, Marzo Edir Da Silva-Grigoletto

**Affiliations:** ^1^ Graduate Program in Physiological Sciences (PROCFIS), Department of Physiology, Federal University of Sergipe, São Cristóvão, Brazil; ^2^ Department of Physical Education, Ages University Center, Paripiranga, Brazil; ^3^ Health Sciences Graduate Program (PPGCS), Department of Medicine, Federal University of Sergipe, Aracaju, Brazil; ^4^ Department of Physical Education, Estácio of Sergipe University Center, Aracaju, Brazil; ^5^ Department of Physiotherapy, Federal University of Sergipe, Lagarto, Brazil; ^6^ Department of Physical Education, Federal University of Sergipe, São Cristóvão, Brazil; ^7^ Laboratory of Biology and Immunology of Cancer and Leishmania, Department of Morphology, Federal University of Sergipe, São Cristóvão, Brazil

**Keywords:** aging, dynapenia, inflammation, exercise, daily living activities, personal autonomy

## Abstract

**Introduction:** Aging can be associated with reduced muscle power, functional decline, and increased plasma concentrations of proinflammatory cytokines. Functional training (FT) can improve muscle power, functional fitness and reduce plasma cytokines. However, the functional training optimal volume required to produce these adaptations must be clarified. Our study analyzed the effects of multiple–set functional training (MSFT) and single–set functional training (SSFT) on postmenopausal women’s muscle power, functional fitness, and inflammatory profile.

**Methods:** Forty–three women were randomly allocated into three groups: multiple–set functional training (*n* = 16, age 64.13 ± 5.17), single–set functional training (*n* = 14, age 63.79 ± 4.88), and control group (CG, *n* = 13, age 64.62 ± 5.44). The bench press and squat exercises evaluated upper and lower limb muscle power. The following tests assessed functional fitness: putting on and taking off a T–shirt, gallon–jug shelf–transfer, standing up and walking around the house, five times sit–to–stand, and 400–m walk. Plasma cytokine (TNF–α, IL–6, and IL 10) concentrations were measured by flow cytometry.

**Results:** Single–set functional training and multiple–set functional training increased upper and lower limbs muscle power and improved functional fitness, except for the putting on and taking off a T–shirt test. Multiple–set functional training reduced TNF–α and IL–6, while single–set functional training reduced only TNF–α. IL–10 was unaffected by exercise.

**Discussion:** Single–set functional training and multiple–set functional training, therefore, promoted similar muscle power and functional fitness improvements over 24 weeks. Multiple–set functional training was more effective than single–set functional training, reducing both TNF and IL–6, while single–set functional training only decreased TNF–α.

## 1 Introduction

Although aging is a natural process, it can be accompanied by some deleterious effects on functional fitness, with a reduction in physical capacities such as muscle power and cardiorespiratory capacity (Picca et al., 2018; Fragala et al., 2019). Especially when associated with physical inactivity, aging is related to an increase in plasma concentration of pro-inflammatory cytokines, a condition known as inflammaging, which may be related to the emergence or maintenance of several chronic diseases (Aiello et al., 2019; Flynn et al., 2019).

Aerobic and strength training and their combination (i.e., concurrent or combined training) are non-pharmacological approaches that can attenuate the deleterious aging effects (Garatachea et al., 2015). Regular physical training promotes increases in muscle strength (Vlietstra et al., 2018), power (Straight et al., 2016), cardiorespiratory fitness (Hurst et al., 2019), and quality of life (Awick et al., 2017; Campbell et al., 2021). Additionally, it reduces pro-inflammatory cytokines plasma concentration (Sardeli et al., 2018).

Functional Training (FT) uses resistance training with other stimuli, such as dynamic balance, motor coordination, flexibility, muscle power, and cardiorespiratory conditioning to increase the individual’s ability to perform activities of daily living (ADLs) safely and efficiently (Da Silva-Grigoletto et al., 2020; La Scala Teixeira et al., 2017).

A FT program can be an excellent approach for all older population, however, in the present study we chose post-menopausal women for some important reasons. Among them, the greater female representation in the older population. In 2030 women will comprise approximately 2/3 of older adults (He et al., 2016). Also, Alexandre et al. (2012) pointed out that older women are more susceptible to a decline in functional fitness. Another important aspect is reducing circulating hormones after menopause, mainly estrogen. This reduction is related to increased circulation of pro-inflammatory cytokines, such as interleukin-1 (IL-1), interleukin-6 (IL-6), and tumor necrosis factor (TNF-α) (Pfeilschifter et al., 2002). Concomitantly, pro-inflammatory cytokines could be a worse effect compromising postmenopausal women’s health and quality of life (Pfeilschifter et al., 2002; Monteiro Santiago et al., 2022).

The ideal dose of FT for postmenopausal women is not fully understood, as there are several variables to be considered for prescription, such as frequency, volume, and intensity. With reference to strength training dose, the current position of the National Strength and Conditioning Association (Fragala et al., 2019) recommends that older women perform between 1-3 sets per exercise and 1-2 multi-joint exercises for the main muscle groups. The intensity should range between 70%–85% of one maximum repetition (1RM). When maximum concentric speed is applied, the intensity should vary between 40%–60% of 1RM. A frequency of 2–3 times a week should be applied using a total of 8–10 multi-joint exercises per session, with the primary objective of stimulating the different components of physical fitness.

Based on the current guidelines, our research group showed increased functional fitness, from different strength parameters (i.e., isometric and dynamic strength) until functional tests similar to the ADLs (Aragão-Santos et al., 2019; Vasconcelos et al., 2020). Also, using FT, resistance training, and bodyweight training, we identified a reduction in pro-inflammatory cytokines (i.e., IL-6 and TNF-α) (Vasconcelos et al., 2020; Monteiro et al., 2022).

Despite the FT and other modalities’ benefits, there is no consensus about the optimal dose of exercise to promote the exercise’s benefits. With respect to resistance training volume, some studies pointed out the superior effects of multiple sets to increase muscle strength and improve body composition (Peterson et al., 2011; Borde et al., 2015). On the other hand, other studies have shown similar effects between single and multiple sets for this same outcome (Galvão and Taaffe, 2005; Radaelli et al., 2018; Cunha et al., 2020). To our best knowledge, no study has evaluated the effect of different FT volumes on functional fitness, muscle power and plasma cytokine concentrations in postmenopausal women.

Thus, we compared different FT volumes on postmenopausal women’s muscle power, functional fitness, and pro-inflammatory cytokines. Our initial hypothesis was that single-set (SSFT) and multiple-set (MSFT) protocols would induce similar effects on muscle power and functional fitness in the twelfth first weeks of training. After that, the MSFT would induce larger adaptations and reduces pro-inflammatory cytokines.

## 2 Materials and methods

### 2.1 Study design

**FIGURE 1:**
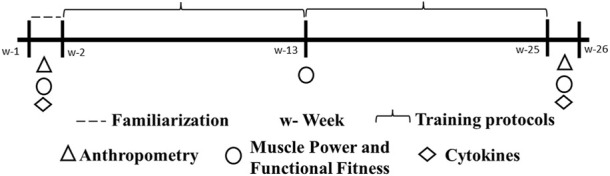
Experimental design.

A 26-week randomized controlled clinical trial was performed with 24 weeks of training (i.e., SSFT and MSFT) and 2 weeks for data collection of the dependent variables (i.e., functional fitness and blood samples) (Figure 1). This study followed the recommendations proposed by CONSORT (Schulz et al., 2010). Also, it was approved by the Research Ethics Committee (nº 2.947.316) and the Brazilian Registry of Clinical Trials (RBR-89KCHG).

### 2.2 Sample

The participants were recruited through leaflets, social media, and advertising. To be eligible to participate in the study, the participants should meet the following criteria: 60 years or older; do not practice any regular physical exercise in the 6 months before the beginning of the study; present a medical certificate indicating health requirements to perform physical activities. Also, the participants with the following conditions were excluded before the start of the training sessions: hypertension ≥ stage 2 (systolic ≥ 160 mmHg and diastolic ≥ 100 mmHg); cognitive impairment; musculoskeletal disorders that do not allow the practice of high-intensity exercises. All participants were informed about ethical standards, objectives, procedures, and risks related to the study and, after acceptance, signed the free and informed consent form.

**FIGURE 2:**
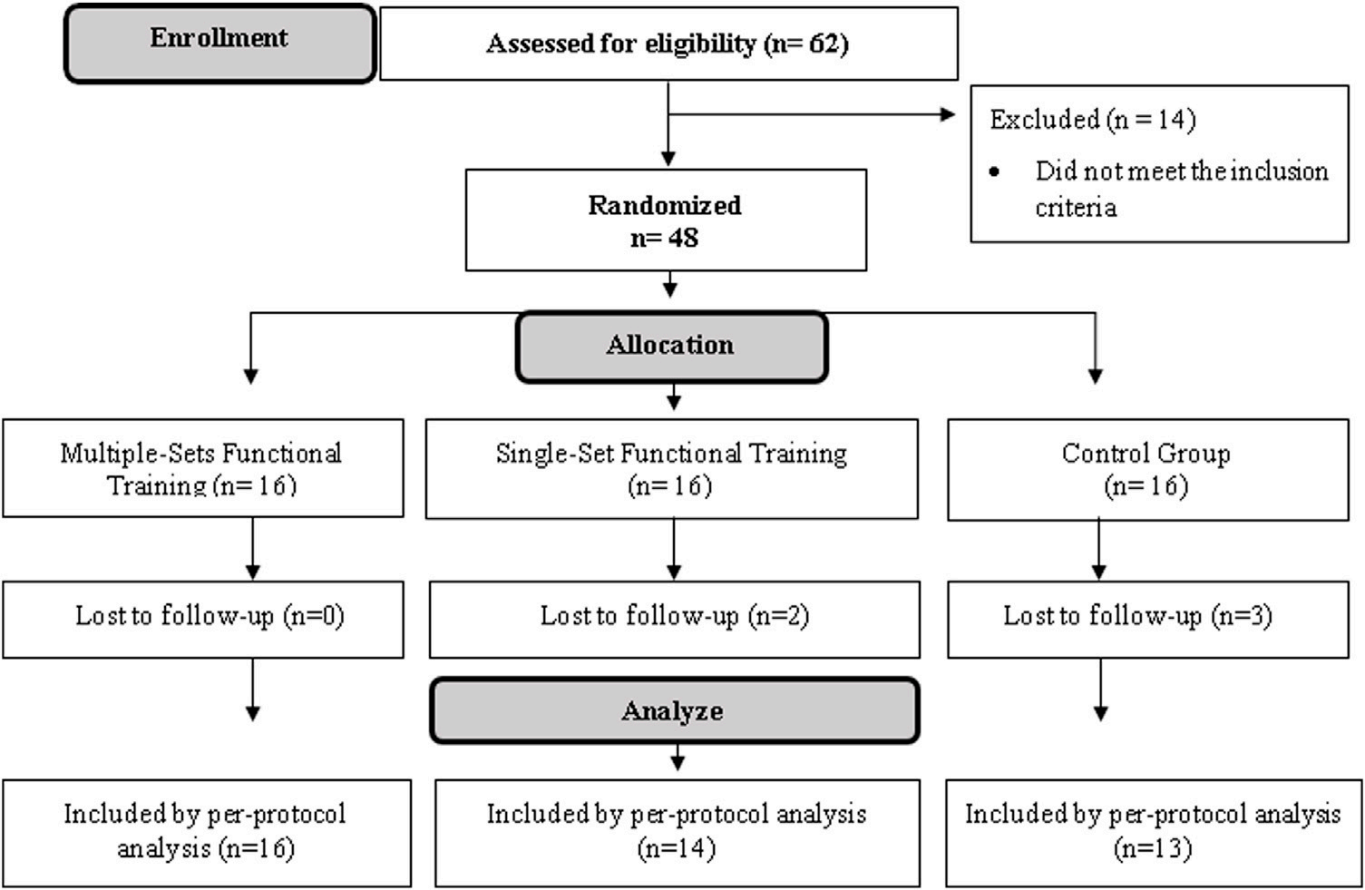
Flow diagram.

Thus, 48 older women were randomly allocated to the Single-Set Functional Training (SSFT: *n* = 16), Multiple-Set Functional Training (MSFT: *n* = 16), and Control Group (CG: *n* = 16). The randomization was based on lower limb power values and the attribution of random numbers for each participant (Microsoft Office^®^ 2016, Washington, United States). Thus, according to the random number, the participants were allocated to each group. A blinded independent researcher performed the allocation (Figure 2).

### 2.3 Exercise protocols

Following three familiarization training sessions, participants in the SSFT and MSFT groups underwent 72 training sessions conducted three times a week on non-consecutive days. The participants performed the exercises according to individual capacity. The effort was monitored and normalized during and after each workout defined by the OMNI-GSE scale (Da Silva-Grigoletto et al., 2013).

All participants performed specific exercises for their daily activities. Each session was divided into four parts: (1) mobility for the main joints (ankle, hip, and glenohumeral) and general warm-up exercises, including ten repetitions of squats and jumps lasting 5 min for SSFT and 10 min for MSFT; (2) intermittent activities, organized in a circuit stimulating agility, coordination, and muscle power (exercises: going up and down a step, alternating waves (rope), skipping, medicine ball throwing, moving between cones and jumping jacks) (OMNI-GSE: 6-7) lasting 6 min for SSFT and 12 min for MSFT; (3) multi-joint exercises organized in a circuit stimulating strength for the lower and upper limbs and intense spine stabilization (exercises: kettlebell lifting, suspension band row, 40 cm sit-up and stand-up, bilateral farmer’s walk, elasticized rowing, front floor plank, and pelvic lift) (OMNI-GSE: 7-8) lasting 8 min for SSFT and 16 min for MSFT.

In parts 2 and 3, the participants performed the exercises at maximum concentric speed. In all sessions, experienced physical education professionals supervised the participants to ensure the proper technique and safety. After the eighteenth session, a training progression was applied, so the external load was incremented in some exercises. In the exercises performed with their body mass, the participants were instructed to change the execution of the movement to achieve the proposed volume (i.e., 8 to 12 repetitions in each exercise). The training density was 30 s of work per 30 s of transition/recovery between stations.

In the 4th part (intermittent exercises), the participants performed a walk at a maximum speed in a 15 m course. Specifically, participants were separated into rows facing each other, with a distance of 15 m between them. The volunteers walked at maximum speed for 15 m and rested while the other participants performed the exercise. The total volume was 8–12 sprints per subject. This part lasted 3 min for SSFT and 6 min for MSFT. After the eighteenth session, the participants ran at maximum speed instead of walking.

Participants in the control group (CG) performed two sets of 15 s of static stretching per exercise (Nelson et al., 2007) for some body regions, such as the neck, shoulders, chest, back, arms, wrists, hands, hips, glutes, anterior thighs, posterior thighs, calves, and feet. All the exercises were performed with a submaximal range of motion and without great physical effort. A frequency of 3 weekly sessions and an average duration of 45 min per session was applied.

The exercise program followed conceptual aspects suggested by Resende-Neto et al. (2016) that were recently tested in some randomized controlled clinical trials (Aragão-Santos et al., 2019; Resende-Neto et al., 2019a; Vasconcelos et al., 2020). In addition, we added detailed descriptions of the exercises and progressions as Supplementary Material S1.

The training load was calculated using the following equation: rate of perceived exertion (RPE) x session duration in minutes (Falk Neto et al., 2020). Specifically, since we used an acceptable range of RPE, we have each group’s training load for the lower and upper limits of the RPE accepted. The only variable that differed between groups was the number of sets (SSFT = 1 and MSFT = 2). The number of exercises (14), repetitions range (8–12), and intensity (6-7 OMINI-GSE scale) were the same for both groups (Table 1).

**TABLE 1:** Functional training variables.

Variables	SSFT	MSFT
Frequency	3-times week	
Format	Circuit training	
Intensity	6–8 OMNI-GSE	
Number of multi-component exercises	6 exercises	
Number of strength/power exercises	8 exercises	
Number of repetitions	8–12	
Contraction velocity	Maximum concentric speed	
Density	1:1	
Number of sets	1 set	2 sets
Interval between exercises	30 s	
Session duration	22 min	44 min
Minimum training load	672 A.U.	1344 A.U.
Maximum training load	1176 A.U.	2352 A.U.

Note: SSFT, single-set functional training; MSFT, multiple-set functional training; OMNI-GSE, perceived exertion scale; s, seconds; min, minutes; A.U., arbitrary unit.

### 2.4 Collection procedures

The functional fitness tests were applied before, after 12 weeks, and after 24 weeks of intervention. The tests were distributed over two consecutive days to minimize interference and with an interval of 2 days from the last training session after the 24 weeks of intervention. Besides the functional tests, before and post 24 weeks, blood samples were collected to measure cytokine plasma concentrations. Specifically, the blood samples were obtained at least 48 h before the first training session. After 24 weeks, the collection was made 1 week after the last training session to avoid bias in the basal cytokine levels. We emphasize that the evaluators were blind to the intervention performed by the participants.

#### 2.4.1 Anthropometry

Body weight was assessed using a clinical scale (Filizola^®^, São Paulo, Brazil), with a maximum capacity of 150 kg. The height (cm) was measured with a stadiometer (Sanny, ES 2030, São Paulo, Brazil).

#### 2.4.2 Muscle power

The muscle power was measured during the pushing and squatting actions performed in a Smith machine with three fixed external loads [Push (4, 8, and 12 kg) and Squat (10, 20, and 30 kg)]. The repetition velocity was measured using a linear encoder (speedometer) connected to the central unit of a data analysis program (Musclelab^®^, 3050e, Oslo, Norway), which made the conversion from velocity (meters per second) to muscle power (Watts). Initially, the participants warmed up by performing ten repetitions using just the barbel of the smith machine for the pushing action and with 5 kg beside the barbel for the squatting action at a moderate speed. After 3 min, they performed 3-5 repetitions at maximum concentric speed for each mentioned load. The repetition with the highest power produced was chosen as the final result (Lohne-Seiler et al., 2013). These measurements were used systematically in our research group’s studies and showed excellent reliability (Aragão-Santos et al., 2019; Vasconcelos et al., 2020).

#### 2.4.3 Functional fitness tests

##### 2.4.3.1 Putting on and taking off a T-shirt

The evaluator asked the participant to stand up with her arms extended at her sides with a large-sized T-shirt in her dominant hand. At the evaluator’s signal (“Go”), she put on the T-shirt entirely and immediately took it off, returning to the starting position (Vale et al., 2006). The participant performed two attempts with 1 min of rest between them, and the evaluator registered the shortest time for analysis. The participant was asked to perform the test again if she did not wear the T-shirt completely (Vale et al., 2006).

##### 2.4.3.2 Gallon-jug shelf-transfer

This test aims to assess global functionality, emphasizing the upper limbs. The participant needed to move five-gallon jugs (3.9 kg each) from a lower (patella height) to an upper shelf (shoulder height) as quickly as possible. The participant was oriented to stand upright and stay laterally to a bookcase (2.13 m × 1.06 m with adjustable shelves). The evaluator showed how the test should be performed. Next, the participants were oriented to keep their back straight, always use the same hand to hold the gallon jugs, use the lower limbs to help with movement, and in case of discomfort or pain, stop the execution. One trial was made to correct possible mistakes. Two attempts were made to measure the execution time after the evaluator’s command (“prepare, now”) with 2 min of rest between them. We considered the shortest time for analysis and disqualified the attempt if the participant moved more than one gallon at a time or alternated hands to perform the movement (Signorile et al., 2007).

##### 2.4.3.3 Standing up and walking around the house

This test assesses the individual’s agility and dynamic balance, using the functional movement of standing up from a sitting position and walking a certain distance with some changes of direction. Initially, a chair was centrally positioned between two cones at 4 m to the back and 3 m to the side of the chair, one cone positioned to the left and the other to the right. The participant started the test in a sitting position, with both feet off the ground, at the evaluator’s command (“Go!”) she needed to get up and walk to the cone on the right side, surround it, return along the same path, and sit down. Next, the participant needed to remove the feet from the ground, stand up, repeat the procedure for the left side, and finish seated with both feet off the ground. To complete one attempt, the participant should perform the abovementioned tasks twice (Dantas et al., 2014). The participant performed one familiarization and two attempts with 1 min of rest between them, and we recorded the shortest time for analysis (Dantas and Vale, 2004).

##### 2.4.3.4 Five times sit-to-stand

In this test, we measured the participant’s ability to sit on and stand up from a chair as quickly as possible five times. This measure assesses functionality and is associated with the power of the lower limbs and the older person’s ability to react. During five consecutive movements, we instructed the volunteer to sit and stand up from a chair without using their arms. The test was timed from the evaluator’s command “go” until the individual sat down and stood up from the chair five times. Three to five hands-on trials were allowed to facilitate familiarity with the procedure. After this period, three attempts were performed, separated by 1 min each, with the shortest time recorded as the test value (Guralnik et al., 1994; Goldberg, 2012).

##### 2.4.3.5 400-m walk

In this test, we instructed each participant to walk over 20 laps as fast as possible, without running, on a previously delimited 20-m course. The participant made only one attempt, and we recorded the execution time for further analysis (Vestergaard et al., 2009).

### 2.5 Cytokines assessment

Four milliliters of blood were collected by venipuncture to obtain at least 1.5 ml of serum. The blood was distributed into EDTA anticoagulant tubes, sodium citrate tubes, and serum separation accelerator tubes. Samples were centrifuged and frozen at −80°C until thawed for evaluation of immunological mediators. The cytokines serum concentrations were assessed by flow cytometry according to the manufacturer’s protocol to obtain measurements for interleukin (IL)-6, IL-10, and tumor necrosis factor (TNF)-α (Human TH1/TH2 CBA II Cytokine Kit, BD Biosciences^®^, San Diego, CA, United States). Briefly, lyophilized cytokine standards and serum samples were processed and analyzed using the BD FACS Calibur flow cytometer, FL4 channel. Three hundred events were acquired for each cytokine used. Data were analyzed using FCAP software, version 3.0 (BD Biosciences^®^, San Diego, CA, United States). Standard curves for each cytokine were generated using a classic mixture of mediators provided. The concentration in each serum sample was determined by interpolating the corresponding standard curve. The mean value obtained was considered whenever both kits evaluated a given cytokine. All calibration curves have a coefficient of linearity (R2) of 0.98 or greater (in many cases, 0.99). This coefficient ensured the test’s reliability. We found an intra-assay coefficient of variation of 26.2%.

### 2.6 Statistical analysis

We calculated the sample using the G*Power software (Erdfelder, Faul, and Buchner, 1996; Kiel, Germany—version 3.1.9.2) based on Aragão-Santos et al. (2019) results for the lower limb muscle power and cytokine values found by Tomeleri et al. (2018) and Vasconcelos et al. (2020). Specifically, we expected an average 10% increase in muscle power, a minimum 10% reduction in proinflammatory cytokines, used an *α* level of 0.05, and a power (1—β) of 0.80. Thus, we needed 36 volunteers (13 participants for each group) to attend the sample size estimation.

All data were analyzed using the Statistical Package for Social Sciences software (SPSS—version 22). We adopted a significance level of 5% (*p* ≤ 0.05) and expressed the results using means, standard deviation, and percentage of change. Homogeneity was tested using the Levene test. Normally distributed data were analyzed using repeated measures ANOVA (3x2) and Tukey’s multiple comparisons *post-hoc* test. Non-normally distributed data were analyzed using the Wilcoxon signed-rank test for within-group comparisons (pre vs. post) and the Kruskal-Wallis test for Between-groups (i.e., CG vs. SSFT vs. MSFT) comparisons. We used the delta for between-group comparisons (i.e., post-pre values per group). All tests were two-tailed, and we calculated the effect sizes (ES) according to the methodological procedures defined by Cohen (2009). The values were interpreted following: <0.19 insignificant; 0.20–0.49 small; 0.50–0.79 moderate; 0.80–1.29 large; >1.30 very large.

## 3 Results

There were no differences between groups at baseline for anthropometric and other characterization variables (Table 2).

**TABLE 2:** Baseline characteristics of the participants.

Variables	MSFT (*n* = 16)	SSFT (*n* = 14)	CG (*n* = 13)	*p*
Age (years)	64.1 ± 5.2	63.8 ± 4.8	64.6 ± 5.4	0.916
Weight (kg)	62.2 ± 14.1	62.7 ± 11.3	65.0 ± 7.40	0.812
Height (m)	1.51 ± 0.04	1.52 ± 0.07	1.50 ± 0.04	0.674
BMI (kg/m^2^)	26.9 ± 5.0	27.2 ± 4.2	28.6 ± 2.3	0.531
5STS (s)	7.32 ± 1.85	8.16 ± 1.05	8.37 ± 1.15	0.116

Note: SSFT, single-set functional training; MSFT, multiple-set functional training; CG, control group; BMI, body mass index; 5STS, five times sit-to-stand. Values in mean and standard deviation (M ± SD).

The average participation rate was 85% (∼62 sessions) in MSFT and 95% (68 sessions) in SSFT.

Both groups increased upper limb power, but only the MSFT significantly improved (post-12 and post-24) compared to pre-intervention values in the bench press (4 kg). Respecting lower limb power, both MSFT and SSFT groups had significant improvement (post-12 and post-24) compared to pre-intervention for squat (10 kg). There were no significant differences between the groups (Table 3).

**TABLE 3:** Effect of Single-Set Functional Training (SSFT) and Multiple-Set Functional Training (MSFT) on upper and lower limb muscle power.

A			Pre	Post 12	Post 24			Pre	Post 12	Post 24	
Group						*p*-value					*p*-value
	Bench press (W) 4 kg	Mean ± SD	31.10 ± 9.49	30.03 ± 9.48	29.81 ± 7.3	Inter. 0.0199	Squat (W) 10 kg	57.63 ± 14.59	54.98 ± 16.93	55.62 ± 17.14	Inter. 0.0016
CG		CI 95%	24.31–37.89	23.24–36.82	24.52–35.10			47.19–68.07	42.87–67.09	43.67–67.88	
		Δ% | ES		−3.44 | 0.02	−4.13 | 0.01				−4.59 | −0.19	−3.48 | −0.11	
		Mean ± SD	32.2 ± 8.96	34.1 ± 8.65	34.5 ± 8.98	Time 0.0406		60.51 ± 11.90	64.21 ± 11.3*	67.08 ± 12.1*	Time 0.0008
SSFT		CI 95%	27.06–37.40	29.05–39.05	29.35–39.72			53.64–67.38	57.67–70.74	60.06–74.09	
		Δ% | ES		+5.90 | 0.21	+7.14 | 0.25				+6.11 | 0.39	+10.85 | 0.67	
		Mean ± SD	33.48 ± 9.00	36.46 ± 9.0*	37.62 ± 9.3*	Group 0.2905		57.19 ± 13.26	59.16 ± 16.1*	64.91 ± 16.7*	Group 0.1203
MSFT		CI 95%	28.67–38.28	31.68–41.24	32.67–42.56			50.13–64.26	50.55–67.77	55.99–73.83	
		Δ% | ES		+8.90 | 0.32	+12.36 | 0.56				+3.44 | 0.16	+13.49 | 0.61	
	Bench press (W) 8 kg	Mean ± SD	44.20 ± 8.70	42.04 ± 8.11	40.41 ± 8.08	Inter. 0.6159	Squat (W) 20 kg	89.71 ± 20.21	84.51 ± 15.88	85.23 ± 17.62	Inter. 0.7049
CG		CI 95%	34.23–45.77	36.23–47.85	34.63–46.19			68.25–97.17	73.15–95.87	72.62–97.84	
		Δ% | ES		−4.88 | 0.04	−3.87 | −0.23				−5,79 | −0.12	+3.40 | −0.03	
		Mean ± SD	45.9 ± 15.83	49.3 ± 16,36	50.5 ± 15,58	Time 0.3504		105.0 ± 26.16	110.9 ± 27.27	110.8 ± 27.05	Time 0.0086
SSFT		CI 95%	36.75–55.03	39.90–58.79	41.48–59.47			89.85–120.1	95.10–126.6	95.18–126.4	
		Δ% | ES		+7.40 | 0.26	+10.02 | 0.36				+5.61 | 0.27	−0,09 | 0.27	
		Mean ± SD	51.63 ± 12.7	54.73 ± 15.0	58.02 ± 16.3	Group 0.0861		112.3 ± 28.68	117.2 ± 26.2	120.0 ± 28.2	Group 0.0084
MSFT		CI 95%	44.86–58.39	46.74–62.72	49.33–66.77			97.03–127.6	103.1–131.2	105.0–135.1	
		Δ% | ES		+6.00 | 0.27	+12.37 | 0.52				+4.36 | 0.22	+6.85 | 0.33	
	Bench press (W) 12 kg	Mean ± SD	56.56 ± 16.9	55.04 ± 14.6	54.53 ± 14.7	Inter. 0.4288	Squat (W) 30 kg	124.0 ± 17.84	121.6 ± 14.28	127.2 ± 10.48	Inter. 0.2788
CG		CI 95%	41.46–65.66	44.57–65.51	43.96–65.10			105.2–142.7	106.6–136.6	116.2–138.2	
		Δ% | ES		−2.68 | 0.01	−0.92 | −0.02				−1.93 | −0.18	+2.58 | 0.24	
		Mean ± SD	55.0 ± 15.41	58.5 ± 17.16	61.5 ± 17.44	Time 0.0001		129.5 ± 31.53	135.7 ± 30.15	141.2 ± 27.47	Time 0.0004
SSFT		CI 95%	49.09–60.82	51.94–65.00	54.82–68.09			111.3–147.7	118.3–153.1	125.3–157.0	
		Δ% | ES		+6.36 | 0.26	+11.81 | 0.48				+4.78 | 0.25	+9.03 | 0.48	
		Mean ± SD	57.19 ± 13.3	59.16 ± 16.2	64.9 ± 16.7	Group 0.8004		137.82 ± 32.60	149.9 ± 29.63	162.8 ± 33.9	Group 0.0082
MSFT		CI 95%	52.49–61.90	53.43–64.89	58.97–70.84			125.8–164.9	134.1–165.7	144.7–180.9	
		Δ% | ES		+3.44 | 0.15	+13.49 | 0.61				+3.09 | 0.16	+11.96 | 0.60	

Note: CG, control group; W, Watts; SD, standard deviation; CI, confidence interval; ES, effect size. Δ%: Change Percentage *p < 0.05 in comparison to pre-test. Two-way ANOVA, followed by Tukey’s *post-hoc*.

**FIGURE 3:**
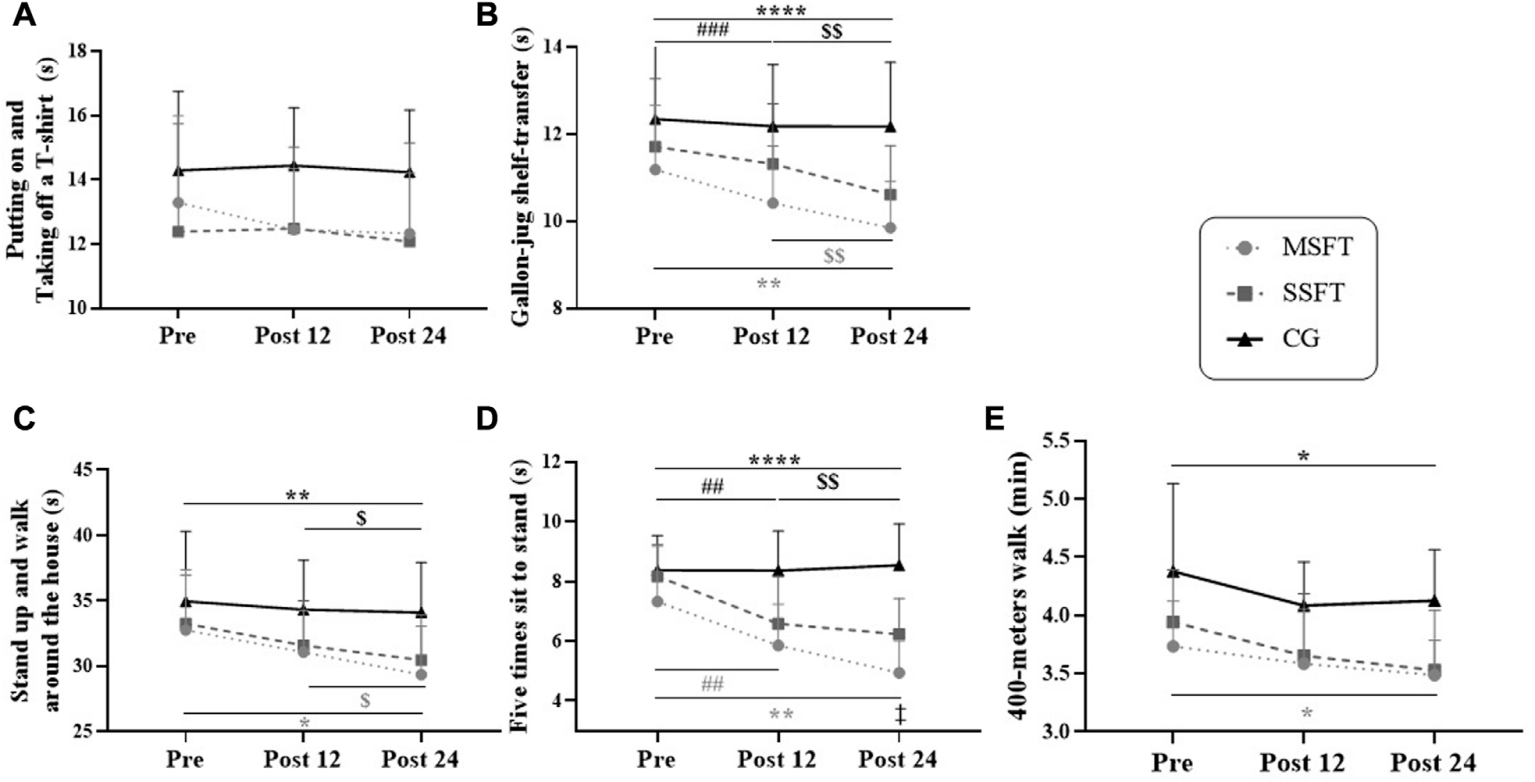
Values observed in functional fitness tests **(A)** putting on and taking off a T-shirt **(B)** gallon-jug shelf-transfer **(C)** standing up and walking around the house **(D)** five times sit-to-stand **(E)** 400-m walk) regarding the control group (CG, *n* = 13, black line with triangles), single-set functional training (SSFT, *n* = 14, grey dashed line with square), and multiple-set functional training (MSFT, *n* = 16, grey dotted line with circles) analyzed by repeated-measures ANOVA with two factors (time × group) and Tukey’s multiple comparisons. Note. Values expressed as mean and standard deviation; differences observed adopting *p* 0.05: ^*^post24 vs. pre; ^$^ post24 vs. post12; ^#^post12 vs. pre; ^‡^ SSFT vs. MSFT. When p < 0.01: ^**^; ^$$^; ^##^. *p*< 0.001: ^***^; ^###^. *p* < 0.0001: ^****^.

Between the MSFT and the SSFT, we detected a difference in the five times sit-to-stand test post 24 weeks of intervention for the MSFT compared to the SSFT (Figure 3D, *p* = 0.0117, ES: 0.29), however, training groups had no differences in the other functional tests. We identified only differences for time comparisons or compared to the CG. We did not find any effect in The Putting on and Taking off a T-Shirt test (Figure 3A).

We detected performance improvements in several functional tests in the MSFT group. Specifically, for the Gallon-jug shelf-transfer (Figure 3B), there was a time effect between pre vs. post-12, *p* = 0.0003, ES: 0.52; post-12 vs. post-24, *p* = 0.0089, ES: 0.43; pre vs. post-24, *p* < 0.0001, ES: 0.90. Also, we detect a time*group interaction effect with differences between MSFT post-12 vs. CG post-12, *p* = 0.0052, ES: 0.82; and MSFT post-24 vs. CG post-24, *p* = 0.0003, ES: 0.90). In the Standing up and walking around the house test (Figure 3C), we found a time effect for the MSFT between post-12 vs. post-24, *p* = 0.0113, ES: 0.53; pre vs. post-24, *p* < 0.0070, ES:0.73. Besides, we detected a time*group interaction effect between MSFT post24 vs. CG post24, *p* = 0.0149, ES: 0.81). For the test Five times sit-to-stand (Figure 3D), the MSFT had a time effect between pre vs. post-12, *p* = 0.0022, ES: 0.79; post-12 vs. post-24, *p* = 0.0031, ES: 0.65; pre vs. post-24, *p* < 0.0001, ES: 1.28. Also, we found a time*group interaction effect between MSFT post-12 vs. CG post-12, *p* = 0.0001, ES: 0.90; and MSFT post-24 vs. CGpost-24, *p* < 0.0001, ES: 0.98). Finally, for the 400-m walk test (Figure 3E), we noticed a time effect for the MSFT between pre vs. post24, *p* = 0.0188, ES: 0.64. Additionally, we detected a time*group interaction between MFST post-12 vs. CG post-12, *p* = 0.0176, ES: 0.78; and MFST post-24 vs. CG post-24, *p* = 0.0031, ES: 0.89).

In the SSFT group, we detected a time effect in Gallon-jug shelf-transfer (Figure 3B): post-12 vs. post-24, *p* = 0.0007, ES: 0.51; pre vs. post-24, *p* = 0.0022, ES: 0.70. Also, we found a time*group effect between SSFT post-24 vs. CG post-24, *p* = 0.0139, ES: 0.80. For the Standing up and walking around the house test we just detected a time effect (Figure 3C): post-12 vs. post-24, *p* = 0.0484, ES: 0.32; and pre vs. post-24, *p* = 0.0250, ES: 0.47). For the Five times sit-to-stand test, we detected a time and an interaction between time and group effects for the SSFT (Figure 3D): pre vs. post-12, *p* < 0.0001, ES: 1.51; pre vs. post-24, *p* < 0.0001, ES: 1.84; SSFT post-12 vs. CG post-12, *p* = 0.0111, ES: 0.80; and SSFT post-24 vs. CG post-24, *p* = 0.0003, ES: 0.89). Finally, we detected a time and interaction between time and group effects for SSFT in the 400-m walk test performances (Figure 3E): pre vs. post-24, *p* = 0.0326, ES: 0.91; and SSFT post-24 vs. CG post-24, *p* = 0.0158, ES: 0.80).

The *p*-values for two-way ANOVA of the functional tests were: Gallon-jug shelf-transfer (interaction: *p* = 0.0003, time: *p* < 0.0001, group: *p* = 0.0052); Standing up and walking around the house (interaction: *p* = 0.2178, time: *p* < 0.0001, group: *p* = 0.0667); Five times sit-to-stand (interaction: *p* < 0.0001, time: *p* < 0.0001, group: *p* < 0.0001); 400-m walk test (interaction: *p* = 0.6897, time: *p* = 0.0002, group: *p* = 0.0025), and Putting on and Taking off a T-Shirt (interaction: *p* = 0.4032, time: *p* = 0.2220, group: *p* = 0.0619).

**FIGURE 4:**
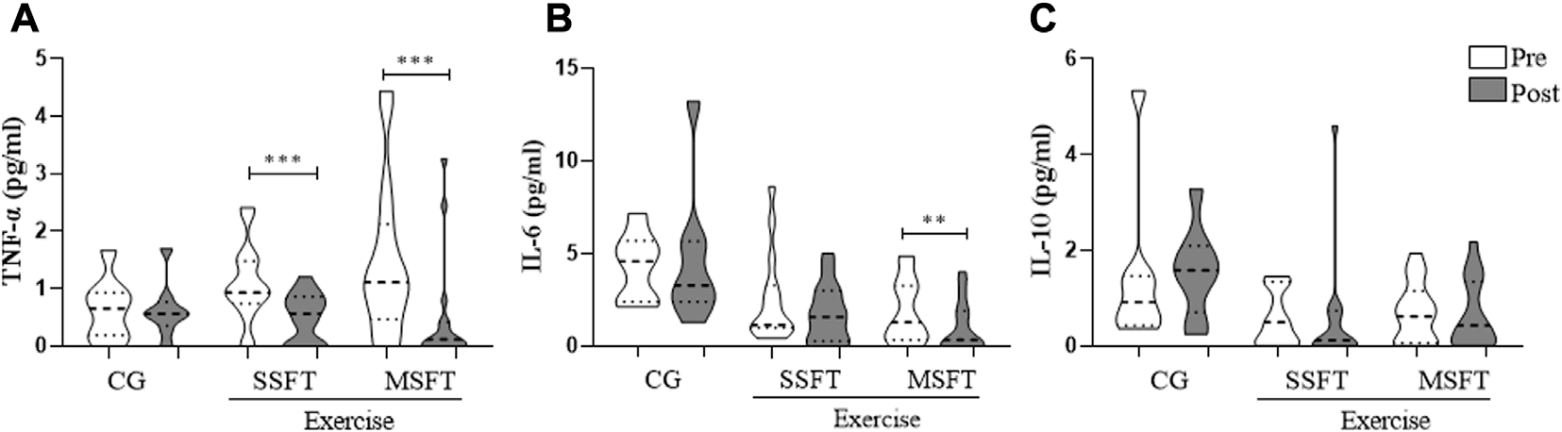
Plasma concentration of cytokines **(A)** TNF-α **(B)** IL-6 **(C)** IL-10 collected pre and post–24 weeks of Single–set functional training (SSFT), Multiple–set functional training (MSFT), or Control (CG). ^*^ *p* < 0.05 in comparison to the pre–test. Within–group comparisons (pre vs. post) based on Wilcoxon signed–rank test. Between–groups (i.e., CG, SSFT, and MSFT) comparisons based on the Kruskal–Wallis test. When *p* < 0.01: ^**^; *p* value < 0.001: ^***^.

We detected a time effect with a reduction in the MSFT for concentrations of TNF-α (*p* = 0.0002; ES: 0.70; Δ%: 66%) and IL-6 (*p* = 0.0026; ES: 0.51; Δ%: 45%). While the SSFT group only reduced concentrations of TNF-α (*p* = 0.0010; ES: 0.84; Δ%: 54%). We found no effect for IL-10 (MSFT, *p* = 0.8928, ES: 0.04, Δ%: 3.8%; SSFT, *p* = 0.3301; ES: 0.06, Δ%: 6.1%) (Figure 4).

## 4 Discussion

The present study compared the effects of SSFT and MSFT on muscle power, functional fitness, and plasma cytokine concentrations in postmenopausal women over 24 weeks. The main finding was that both training groups produced a similar increase in muscle power and functional capacity after 12 weeks of intervention. Also, the MSFT promoted additional benefits after 24 weeks. These findings corroborated our initial hypothesis. Furthermore, it is the first study to assess different FT volumes’ effects on pro-inflammatory cytokines. Interestingly, our results demonstrated that SSFT and MSFT reduced TNF-α plasma concentration, and only the MSFT group decreased IL- 6.

Muscle power declines faster than maximal strength during aging, and this reduction contributes to lower functional fitness in older people (Byrne et al., 2016). In the present study, both intervention groups increased upper and lower limb muscle power. We only found a significant improvement in pushing (MSFT—bench press 4 kg) and squatting actions (MSFT and SSFT—squat 10 kg) at the lowest loads evaluated. One possible reason for this finding could be the functional training protocol, since part 2 (muscle power-oriented) was performed with smaller external loads or just the body mass. Additionally, the results produced by the MSFT could be derived from the higher volume applied to improve the upper limb power, since most of the training was focused on lower limb activities. SSFT and MSFT have common characteristics, such as multi-component work and maximum concentric speed, that may explain positive adaptations over time in lower limb muscle power (Ramírez-Campillo et al., 2014; Cadore and Izquierdo, 2018).

Our study is the first to compare the effects of different volumes of functional training on postmenopausal women. Our findings, however, corroborate other studies investigating different volumes’ effects using resistance training. Several articles indicated that both training protocols improved muscle function in postmenopausal and older women (Galvão and Taaffe, 2005; Abrahin et al., 2014) even though the larger volume group experienced greater improvements in specific functional tests.

We found no improvements in the upper limb’s functional fitness. One explanation could be related to the number of exercises focused on the upper limbs corresponding to 30% of the session. We designed the training protocol with a larger number of exercises for the lower limb because they are more affected by aging than the upper limbs (Larsson et al., 1979; Rikli and Jones, 2013). Thus, applying a greater volume of exercises for the upper limb could increase muscle strength and power. Additionally, according to Dantas et al. (2014) classification, our baseline values in the Putting on and Taking off a T-shirt test are classified as regular and good. Then, a greater stimulus should be applied to improve the performance in this test since our good initial participant’s level.

Although SSFT improved the Five times sit-to-stand test performance, the MSFT was superior, like previously published results (Ribeiro et al., 2015; Barbalho et al., 2017). The improvement in both groups could be attributed to the specificity of the squatting action and the higher number of lower limb exercises. Also, both groups performed the exercises at maximum concentric speed, stimulating muscle power (Byrne et al., 2016; Straight et al., 2016). Thus, likely the training stimuli increased the activation of type II muscle fibers, the excitability of alpha motor neurons in the spinal cord, and the recruitment and synchronization of motor units promoting more efficient adaptive responses in muscle power (Van Roie et al., 2020).

Both experimental groups significantly improved agility, coordination, dynamic balance, and global functionality, corroborating the current literature (Barbalho et al., 2017; Radaelli et al., 2018). The multi-component characteristic of our training protocol could explain the observed improvements in the functional fitness measures similar to daily activities (Resende-Neto et al., 2019b). In addition, regular exercise practice is associated with neural adaptations, such as increased motor unit recruitment, neural firing rate, and motor unit synchronization, promoting better performance the functional tests (Carroll et al., 2001; Kraemer and Ratamess, 2004; Lesinski et al., 2015; Schott et al., 2019; Wolf et al., 2020).

We found improvements for both training groups in aerobic endurance based on the 400-m walk test performance. The improvements were likely derived from the neuromuscular and metabolic training stimulus, such as the circuit-training session and the interval running in the last training part. Our findings corroborate Resende-Neto et al. (2016), which reported an 8% increase in the cardiorespiratory capacity of older women after a similar FT protocol.

Although physical exercise can reduce pro-inflammatory cytokines, there is no consensus about postmenopausal women (Macêdo Santiago et al., 2018; Sardeli et al., 2018; Flynn et al., 2019). For instance, Prestes et al. (2018) did not observe changes in circulating levels of IL-4 after 16 weeks of RT. IL-4 is an anti-inflammatory cytokine that plays an important role promoting muscle hypertrophy and antagonizes the activity of pro-inflammatory cytokines such as IL-6, TNF-α and IL-1β. Another study pointed out a reduction for C-reactive protein and TNF-α but not for IL-6 after 12 weeks of RT (Phillips et al., 2012). Similarly, Monteiro et al. (2022) found a reduction in TNF-α concentration induced by RT and bodyweight training after a 24-week intervention, with only the BWT decreasing IL-6.

Our findings corroborate Vasconcelos et al. (2020), that found an FT-induced reduction in TNF-α and IL-6 concentrations after 24 weeks of intervention. In the present trial, single-set training reduced TNF-α, a cytokine involved in the process of muscle breakdown and production of other cytokines, such as IL-6, that play a vital role in weakening muscle mass, strength, and power (Pfeilschifter et al., 2002; Vasconcelos et al., 2020).

It should be noted, however, that during and shortly after exercise, due to muscle contraction, IL-6 is produced and released by the muscle independently of TNF-α (Nieman et al., 1985; Forti et al., 2016). Acutely, increased IL-6 promotes higher hepatic glucose production during exercise, lipolytic action in skeletal muscle, regulation of insulin and glucose uptake by the muscle cell (Febbraio et al., 2004; Pedersen et al., 2007; Pedersen and Febbraio, 2012). Besides, IL-6 reduces low-grade chronic inflammation by stimulating inhibitory factors that limit the production of pro-inflammatory cytokines (e.g., IL-1 β and TNF-α) and stimulate the production of anti-inflammatory cytokines (e.g., IL-10) (Gleeson et al., 2011; Forti et al., 2016). Thus, muscle-derived IL-6 is associated with a lower risk of chronic diseases and premature deaths over time (Pedersen and Febbraio, 2012). Therefore, to avoid the possible acute effects of exercise on basal cytokines concentrations, we evaluated the cytokines only 1 week after the end of the exercise protocol.

In this study, only MSFT reduced IL-6. The greater training volume and, consequently, the higher muscle-derived IL-6 concentration during and after training could have generated an anti-inflammatory environment. The already mentioned muscle-derived IL-6 effects in response to exercise support this hypothesis. Also, a meta-analysis with older diabetic adults pointed out that longer programs or a higher number of sessions demonstrated larger IL-6 reductions (Hayashino et al., 2014). Another possible explanation for the IL-6 declines could be associated with body composition changes since muscle hypertrophy, or lower fat mass could favor the reduction of these pro-inflammatory markers (Sardeli et al., 2018). We did not evaluate these variables, which is a limitation of our study. We pointed out, however, reductions in pro-inflammatory cytokines, an important finding related to the quality of life and reduced morbidity (Flynn et al., 2019). Much like Vasconcelos et al. (2020), we found no modulation of this cytokine after a TF protocol. IL-10 modulation is a controversial result, with other studies also demonstrating no differences in concentration after training programs (Campo et al., 2015).

Our study provides an important practical application, since time constraints had been reported as one of the main barriers to exercise adherence (Schutzer, 2004; Aily et al., 2017). Besides, high-volume protocols were associated with higher abandonment rates (Hass et al., 2000). Thus, an effective time-efficient protocol using a single set approach could be an excellent strategy to promote benefits in the functional fitness of postmenopausal women. Furthermore, SSFT can be applied to community programs, as it allows more participants per hour of intervention.

## 5 Conclusion

SSFT and MSFT promote similar muscle power and functional fitness improvements over 24 weeks. Only MSFT reduces TNF-α and IL-6, while SSFT only decreases TNF-α levels. These findings can help professionals in choosing the training volume for postmenopausal women.

## Data Availability

The raw data supporting the conclusion of this article will be made available by the authors, without undue reservation.
