# Supplementary material for: A single-set functional training program increases muscle power, improves functional fitness, and reduces pro-inflammatory cytokines in postmenopausal women: A randomized clinical trial
Source: Front Physiol. 2023 Mar 22;14:1054424. doi: 10.3389/fphys.2023.1054424 (PMC10075356; doi:10.3389/fphys.2023.1054424)
Supplement: Supplementary file 1 [file Table1.DOCX]

| **SSFT** | **Part 1** | **Part 2** | | **Part 3** | | **Part 4** |
| --- | --- | --- | --- | --- | --- | --- |
|  | Preparation for movement with mobility exercises for the cervical, shoulder, thoracic, hip and ankle joints | **1-18 session** | **18-36 session** | **1-18 session** | **18-36 session** | Intermittent activity through interval running |
|  |  | Step up and down - Front view | Step up and down - Side | Deadlift with Kettlebell | Kettlebell deadlift (20% load increase) |  |
|  |  | *Alternating waves (rope) -* Linear | *Alternating waves (rope) - Side* | Row on the trx (Neutral) | Trunk Rowing (Pronade) |  |
|  |  |  |  | Sit and stand on the 40 cm bench | Squat with kettlebell |  |
|  |  | Medicine ball throws on the ground (Slam ball 5 kg) | Medicine Ball Wall Throws  (Slam ball 5 kg) | Horizontal adduction with elastic | 60 cm bench push-ups |  |
|  |  |  |  | Single-sided Farmers Walk – Light Load (Kettlebells) | Farmers walk Unilateral – Light load (Kettlebells) |  |
|  |  | Front offset between cones (zig-zag) | Lateral displacement between cones (zig-zag) | Bungee Row (Neutral) | Bungee Row (Pronade) |  |
|  |  |  |  | Bilateral pelvic lift | Bilateral pelvis lift – 10kg |  |
|  |  | Agility Ladder - Front Plane | Agility ladder - Lateral plane | Front board on the 40 cm bench | Front plank on the ground |  |
|  |  | Jumping jack - Front view | Jumping jack - Sagittal plane |  |  |  |
|  | **Total time: 5 min, 3-5 exercises per joint, 1 set of 8 seconds** | **Total time: 6 min, 6 activities, 1 pass, 30 sec per station, 1/1 density. OMINI-GSE: 6 to 7** | **Total time: 6 min, 6 activities, 1 pass, 30 sec per station, 1/1 density. OMINI-GSE: 6 to 7** | **Total time: 8 min, 8 exercises, 1 set of 08-12 reps, 30 sec per station, density 1/1. OMINI-GSE: 6 to 7** | **Total time: 8 min, 8 exercises, 1 set of 08-12 reps, 30 sec per station, density 1/1. OMINI-GSE: 6 to 7** | **Total time: 3 min, 5-8 efforts, 1/1 density and OMINI-GSE scale from 7 to 8.** |
|  | Preparation for movement with mobility exercises for the cervical, shoulder, thoracic, hip and ankle joints | **36-54 session** | **54-72 session** | **36-54 session** | **54-72 session** | Intermittent activity through interval running |
|  |  | Jump over step - Frontal | Step jump - Lateral | Kettlebell Deadlift (Unilateral – Central) | DeadLift with Ketlbell (Bilateral – 20 Kg) |  |
|  |  | Alternating waves (rope) with dynamic squats - Linear | Alternating waves (rope) with dynamic squat -2 steps Lateral | Suspension Tape Row (Supinated) | Suspension tape row (Pronation + Neutral) |  |
|  |  | Medicine ball throws on the ground (Medball 3kg reactive) | Medicine Ball Wall Throws (Medball 3kg reactive) | Front sandbag squat | Sandbag shoulder squat |  |
|  |  | Run and jump between cones - Frontal | Side shift by tapping small cones | Push-ups on the 40 cm bench | Push-ups no banco de 30 centímetros |  |
|  |  | Agility Ladder – Jumping Front | Agility Ladder – 1, 2 for side | Two-way Farmers Walk – 20% Charge Increase (Kettlebells) | One-way Farmers Walk – 20% Charge Increase (Kettlebells) |  |
|  |  | Jumping jack -Front plan (1kg weight) | Jumping jacks - Sagittal plane (1kg weight) | One-sided bungee row (Neutral) | Unilateral elastic row (Pronade) + hip flexion |  |
|  |  |  | |  |  |  |
|  |  |  | | Pelvic lift with step | Pelvic lift on the step with a load of 10 kg |  |
|  |  |  |  | Side plank on the ground | Sit and stand up from the ground |  |
|  | **Total time: 5 min, 3-5 exercises per joint, 1 set of 8 seconds** | **Total time: 6 min, 6 activities, 1 pass, 30 sec per station, 1/1 density. OMINI-GSE: 6 to 7** | **Total time: 6 min, 6 activities, 1 pass, 30 sec per station, 1/1 density. OMINI-GSE: 6 to 7** | **Total time: 8 min, 8 exercises, 1 set of 08-12 reps, 30 sec per station, density 1/1. OMINI-GSE: 6 to 7** | **Total time: 8 min, 8 exercises, 1 set of 08-12 reps, 30 sec per station, density 1/1. OMINI-GSE: 6 to 7** | **Total time: 3 min, 5-8 efforts, 1/1 density and OMINI-GSE scale from 7 to 8.** |

**Table S1**: Description of the SSFT group training sessions carried out over 24 weeks.

**Table S2**: Description of the MSFT group training sessions carried out over 24 weeks.

| **MSFT** | **Part 1** | **Part 2** | | **Part 3** | | **Part 4** |
| --- | --- | --- | --- | --- | --- | --- |
|  | Preparation for movement with mobility exercises for the cervical, shoulder, thoracic, hip and ankle joints | **1-18 session** | **18-36 session** | **1-18 session** | **18-36 session** | Intermittent activity through interval running |
|  |  | Step up and down - Front view | Step up and down - Side | Deadlift with Kettlebell | Kettlebell deadlift (20% load increase) |  |
|  |  | *Alternating waves (rope) -* Linear | *Alternating waves (rope) - Side* | Row on the trx (Neutral) | Trunk Rowing (Pronade) |  |
|  |  |  |  | Sit and stand on the 40 cm bench | Squat with kettlebell |  |
|  |  | Medicine ball throws on the ground (Slam ball 5 kg) | Medicine Ball Wall Throws  (Slam ball 5 kg) | Horizontal adduction with elastic | 60 cm bench push-ups |  |
|  |  |  |  | Single-sided Farmers Walk – Light Load (Kettlebells) | Farmers walk Unilateral – Light load (Kettlebells) |  |
|  |  | Front offset between cones (zig-zag) | Lateral displacement between cones (zig-zag) | Bungee Row (Neutral) | Bungee Row (Pronade) |  |
|  |  |  |  | Bilateral pelvic lift | Bilateral pelvis lift – 10kg |  |
|  |  | Agility Ladder - Front Plane | Agility ladder - Lateral plane | Front board on the 40 cm bench | Front plank on the ground |  |
|  |  | Jumping jack - Front view | Jumping jack - Sagittal plane |  |  |  |
|  | **Total time: 10 min, 3-5 exercises per joint, 1 set of 8 seconds** | **Total time: 12 min, 6 activities, 2 pass, 30 sec per station, 1/1 density. OMINI-GSE: 6 to 7** | **Total time: 12 min, 6 activities, 2 pass, 30 sec per station, 1/1 density. OMINI-GSE: 6 to 7** | **Total time: 16 min, 8 exercises, 2 sets of 08-12 reps, 30 sec per station, density 1/1. OMINI-GSE: 6 to 7** | **Total time: 16 min, 8 exercises, 2 sets of 08-12 reps, 30 sec per station, density 1/1. OMINI-GSE: 6 to 7** | **Total time: 6 min, 5-8 efforts, 1/1 density and OMINI-GSE scale from 7 to 8.** |
|  | Preparation for movement with mobility exercises for the cervical, shoulder, thoracic, hip and ankle joints | **36-54 session** | **54-72 session** | **36-54 session** | **54-72 session** | Intermittent activity through interval running |
|  |  | Jump over step - Frontal | Step jump - Lateral | Kettlebell Deadlift (Unilateral – Central) | DeadLift with Ketlbell (Bilateral – 20 Kg) |  |
|  |  | Alternating waves (rope) with dynamic squats - Linear | Alternating waves (rope) with dynamic squat -2 steps Lateral | Suspension Tape Row (Supinated) | Suspension tape row (Pronation + Neutral) |  |
|  |  | Medicine ball throws on the ground (Medball 3kg reactive) | Medicine Ball Wall Throws (Medball 3kg reactive) | Front sandbag squat | Sandbag shoulder squat |  |
|  |  | Run and jump between cones - Frontal | Side shift by tapping small cones | Push-ups on the 40 cm bench | Push-ups no banco de 30 centímetros |  |
|  |  | Agility Ladder – Jumping Front | Agility Ladder – 1, 2 for side | Two-way Farmers Walk – 20% Charge Increase (Kettlebells) | One-way Farmers Walk – 20% Charge Increase (Kettlebells) |  |
|  |  | Jumping jack -Front plan (1kg weight) | Jumping jacks - Sagittal plane (1kg weight) | One-sided bungee row (Neutral) | Unilateral elastic row (Pronade) + hip flexion |  |
|  |  |  | |  |  |  |
|  |  |  | | Pelvic lift with step | Pelvic lift on the step with a load of 10 kg |  |
|  |  |  |  | Side plank on the ground | Sit and stand up from the ground |  |
|  | **Total time: 10 min, 3-5 exercises per joint, 1 set of 8 seconds** | **Total time: 12 min, 6 activities, 2 pass, 30 sec per station, 1/1 density. OMINI-GSE: 6 to 7** | **Total time: 12 min, 6 activities, 2 pass, 30 sec per station, 1/1 density. OMINI-GSE: 6 to 7** | **Total time: 16 min, 8 exercises, 2 sets of 08-12 reps, 30 sec per station, density 1/1. OMINI-GSE: 6 to 7** | **Total time: 16 min, 8 exercises, 2 sets of 08-12 reps, 30 sec per station, density 1/1. OMINI-GSE: 6 to 7** | **Total time: 6 min, 5-8 efforts, 1/1 density and OMINI-GSE scale from 7 to 8.** |
